# Comparison of the Accuracy of CBCT and MDCT Images in Measuring the Thickness of the Posterior Footplate of the Middle Ear in Iranian -

**DOI:** 10.31661/gmj.vi.3900

**Published:** 2025-12-16

**Authors:** Sanaz Sharifi Shooshtari, Nader Saki, Zohreh Roozbahani, Kowsar Farahmandfar, Nila Bagheri

**Affiliations:** ^1^ Department of Oral and Maxillofacial Radiology, Faculty of Dentistry, Ahvaz Jundishapur University of Medical Sciences, Ahvaz, Iran; ^2^ Hearing Research Center, Ahvaz Jundishapur University of Medical Sciences, Ahvaz, Iran

**Keywords:** Middle Ear, Footplate Thickness, CBCT, MDCT

## Abstract

**Background:**

Advancements in radiological imaging have transitioned from two-dimensional radiography to three-dimensional cone beam computed tomography (CBCT), offering high spatial resolution with reduced radiation doses compared to multidetector computed tomography (MDCT). While MDCT remains the standard for detailed visualization of bony structures like the ossicular chain, its higher radiation exposure is a concern. This study compares the accuracy of high-resolution (HR) and low-resolution (LR) CBCT with MDCT in measuring the thickness of the posterior footplate of the middle ear to identify a reliable, low-radiation alternative.

**Materials and Methods:**

Twelve adult human temporal bones from Imam Khomeini Hospital’s ENT Department were imaged using HR-CBCT, LR-CBCT (Jundishapur Dental School), and MDCT (Siemens Sensation 64-slice). Standardized imaging protocols ensured reproducibility, with measurements of posterior footplate thickness conducted in axial, coronal, and sagittal planes by two blinded radiologists. Data were analyzed using SPSS v23, with inter-modality agreement assessed via Kappa coefficient and differences evaluated with the McNemar test.

**Results:**

Mean posterior footplate thickness was 2.38 mm (HR-CBCT), 2.37 mm (LR-CBCT), and 2.23 mm (MDCT), with no significant differences (P0.05). HR-CBCT and LR-CBCT showed comparable accuracy to MDCT.

**Conclusion:**

CBCT, particularly HR-CBCT, offers a reliable, lower-radiation alternative to MDCT for otologic imaging, maintaining high resolution for middle ear assessments.

## Introduction

Innovative applications of computed tomography in clinical settings have emerged due to advancements in radiological imaging technology. Techniques have progressed from two-dimensional radiography to three-dimensional (3D) digital imaging [1]. Cone beam computed tomography (CBCT) provides integrated three-dimensional imaging with exceptional spatial resolution. In routine clinical practice, this method is primarily utilized in dental and maxillofacial imaging. Several studies have demonstrated promising outcomes for imaging other regions and finer details [2]. CBCT encompasses a conical field of view (FOV) with a height of a few centimeters and an axial diameter ranging from 4 cm to approximately 20 cm [3]. The FOV can be tailored to the specific examination type in some equipment [4]. The ability to restrict the imaging area to the relevant region is a key benefit of CBCT. This technique may prove valuable in otologic imaging within the head and neck region, as the information required is often localized and unilateral. In treatment planning, accurate imaging of the patient’s middle ear before or after surgery is critical. Frequent radiological examinations are often necessary to conduct preoperative and postoperative evaluations and monitor the patient’s middle ear, particularly in cases of chronic otitis media or cholesteatoma disease. CT has traditionally been the primary tool in otologic imaging due to the requirement for detailed visualization of bony structures, such as the ossicular chain and air-filled spaces in a targeted area [5].

The continuous and cumulative radiation exposure from repeated CT examinations represents a significant drawback. The imaging area is frequently larger than necessary for the assessment, resulting in unnecessary radiation exposure for the patient [6]. Multidetector computed tomography (MDCT) is the standard approach for radiological evaluation of hearing loss. However, this method subjects patients to high radiation doses. The use of CBCT has grown significantly in dental and oral surgery [7], and its application in craniofacial surgery has expanded considerably over the past 15 years [8]. CBCT is associated with a lower radiation dose compared to MDCT [9]. Comparative studies since 2007 have evaluated MDCT and CBCT using human temporal bone specimens or in vitro settings in the follow-up of middle ear prostheses, active middle ear implants, cochlear implants, and bone-borne hearing aids [10]. Findings suggest that the analysis of temporal bone structures using CBCT is satisfactory and is associated with relatively low radiation doses [11]. Thus, this study compares the accuracy of high-resolution and low-resolution CBCT images and MDCT in measuring the thickness of the posterior footplate of the middle ear to establish a reliable method that minimizes radiation dose while maintaining high resolution.

## Materials and Methods

This was a laboratory cross-sectional study on 12 adult human temporal bones with intact overlying soft tissue, obtained from the archive of the dissection room of the ENT Department at Imam Khomeini Hospital in Ahvaz. All specimens were anonymized, cataloged, and handled according to standard anatomical research ethics. The sample size was determined based on previous studies (Dahmani-Causse et al., [12]) and in consultation with a biostatistician using Med-Calc software , targeting a 5% significance level and 80% statistical power. Inclusion criteria required the temporal bones to be structurally intact, free from fractures, surgical alterations, or pathological changes, while exclusion criteria included evidence of prior trauma or congenital deformities.

Imaging of the posterior footplate of the stapes in the middle ear was performed using multiple modalities: high-resolution (HR) and low-resolution (LR) cone-beam computed tomography (CBCT) at Jundishapur Dental School, and multidetector computed tomography (MDCT) at Imam Khomeini Hospital. For MDCT, each temporal bone was placed in a standardized orientation within a plastic container, stabilized using alginate impression material to minimize motion artifacts. MDCT images were acquired using a helical CT scanner (Siemens Sensation 64-slice) and stored in the hospital’s CT workstation software. Acquisition parameters were standardized at 120 kVp, 70 mAs, slice thickness of 0.6 mm, and a pitch of 1.4, optimized for bone and soft-tissue contrast.

For CBCT imaging, temporal bones were carefully secured with adhesive tape in the NewTom VGi CBCT apparatus to ensure reproducible positioning. Images were obtained with a field of view (FOV) of 8 × 12 cm, 110 kVp, and acquisition times of 3.6 seconds for LR scans and 5.4 seconds for HR scans. All CBCT datasets were reconstructed in "denture mode" to enhance visualization of soft tissues and fine bony details and were subsequently stored in NNT software . Images from both MDCT and CBCT modalities were coded, anonymized, and archived on DVD media for blinded evaluation.

Image analysis was performed independently by two experienced observers to reduce measurement bias: an oral and maxillofacial radiologist for CBCT images and a general radiologist for MDCT images. Observers evaluated images in a semi-darkened room on a 14-inch LED monitor (1920 × 1080 resolution) and were allowed to adjust windowing, contrast, and zoom for optimal visualization. The thickness of the posterior footplate was measured digitally in all three anatomical planes (axial, coronal, and sagittal) using the dedicated measurement tools of the CT and CBCT software. Measurements were recorded on pre-prepared data collection sheets, with each observer performing two separate measurements per plane to assess intra-observer reliability. The compiled results were then submitted to a statistician for analysis.

**Figure-1:**
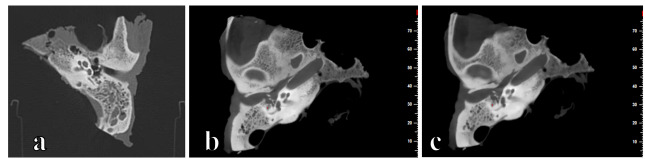
Figure 1. (a) CT image of the posterior footplate of the middle ear; (b) High-resolution and (c) Low-resolution CBCT image of the posterior footplate of the middle ear

Representative images of the posterior footplate obtained using MDCT, HR CBCT, and LR CBCT were prepared (Figures-1) to illustrate imaging quality and anatomical details.

Data analysis was performed descriptively and inferentially using SPSS software (v23, IBM Corp., Chicago, IL, USA). Inter-modality agreement for footplate thickness measurements was evaluated using the Kappa coefficient, while differences in paired measurements were assessed using the McNemar test.

## Results

**Table:** Table1. Thickness of the Posterior Footplate of the Middle Ear and Comparison of Imaging Modalities

Variable / Comparison	N	Mean (mm)	SD	Minimum	Maximum	P-value
CBCT High Resolution	12	3.38	0.64	1.34	3.40	0.79
CBCT Low Resolution	12	2.37	0.64	1.32	3.42	0.80
MDCT	12	2.23	0.54	1.28	2.78	—

The thickness of the posterior footplate of the middle ear was measured using three imaging modalities: high-resolution CBCT, low-resolution CBCT, and MDCT. Descriptive statistics for each modality are presented in Table-1. The mean thickness of the posterior footplate was 2.38 mm (SD=0.64) for high-resolution CBCT, 2.37 mm (SD=0.64) for low-resolution CBCT, and 2.23 mm (SD=0.54) for MDCT. The minimum and maximum values for each modality are also reported in Table-1. Independent samples t-tests were conducted to compare the thickness measurements between the imaging modalities. There was no significant difference between high-resolution CBCT and MDCT, t(11)=-0.28, P=.79, indicating that high-resolution CBCT measurements were comparable to MDCT. Similarly, low-resolution CBCT did not differ significantly from MDCT, t(11)=-0.26, P=.80. Additionally, no significant difference was observed between high-resolution and low-resolution CBCT, t(11) = 0.05, P>.05.

## Discussion

The present study compared multidetector computed tomography (MDCT) and cone-beam computed tomography (CBCT) regarding measurement accuracy of the posterior middle ear footplate to identify an imaging protocol that offers high accuracy, low radiation dose, and clinical practicality. Studies report that MDCT and CBCT are valuable tools for evaluating inner ear structures, particularly for assessing cochlear implant (CI) electrode position and insertion trauma [10][13][14]. MDCT is currently the gold standard for inner ear imaging; however, CBCT offers several advantages, including lower radiation doses and comparable image quality [15][16][17]. In our study, the thickness of the posterior middle ear footplate was measured using MDCT, high-resolution (HR) CBCT, and low-resolution (LR) CBCT. The mean thickness was 2.38 mm (SD=0.64) for HR-CBCT, 2.37 mm (SD=0.64) for LR-CBCT, and 2.23 mm (SD=0.54) for MDCT.

The results revealed that footplate thickness measurements were slightly higher in HR-CBCT compared to MDCT, though this difference was not statistically significant (P=0.79). Similarly, no significant difference was observed between LR-CBCT and MDCT (P=0.80) or between HR-CBCT and LR-CBCT (P>0.05). This equivalence suggests that CBCT, regardless of resolution, provides measurement accuracy comparable to MDCT. The slightly higher mean thickness in HR-CBCT may be attributed to its isotropic voxels, which offer sharper edge definition and improved visualization of fine bony structures. However, the lack of significant difference between HR- and LR-CBCT suggests that LR-CBCT’s resolution is sufficient for accurate footplate measurements, likely due to optimized image reconstruction algorithms in the "denture mode" used in this study.

Previous studies have compared MDCT and CBCT using human temporal bone specimens [13]. For example, Burck et al. conducted a radiohistological study in Germany to assess CBCT and MDCT for cochlear implant imaging, concluding that CBCT provides superior image quality for temporal bone structures, particularly in visualizing cochlear implant electrode placement [20]. While Burck et al. focused on qualitative image quality, our study extends these findings by demonstrating CBCT’s quantitative accuracy in measuring footplate thickness. Similarly, Debeaupte et al. conducted a prospective multicenter study in Germany comparing CBCT and MDCT for temporal bone reconstruction in patients with hearing loss. They reported satisfactory diagnostic agreement (kappa=0.69), supporting CBCT’s reliability for assessing conductive hearing loss [21]. Our quantitative findings align with these results, confirming CBCT’s equivalence to MDCT for precise measurements in otologic imaging.

In another study, Kemp et al. in the United States compared CBCT and MDCT for middle ear imaging, noting that CBCT provided superior visualization of structures such as the facial canal, footplate, and cochlear aqueduct, with image quality comparable to MDCT for other structures like the tegmen tympani [16]. Although Kemp et al. emphasized qualitative visibility, their findings support CBCT’s utility in otologic imaging, consistent with our quantitative results. Similarly, Komori et al. in Japan evaluated total ossicular replacement prosthesis (TORP) placement in six patients using CBCT and MDCT, finding that CBCT better detected TORP displacement and malposition [23]. While their study was qualitative and in vivo, unlike our quantitative ex vivo analysis, it reinforces CBCT’s diagnostic reliability.

Zou et al. in China used HR-CBCT to evaluate temporal bone structures, including the footplate, and reported remarkable detail compared to MDCT [24]. Their qualitative assessment of CBCT’s ability to visualize fine structures supports our finding that CBCT provides accurate measurements, even at lower resolution. Similarly, Dahmani-Causse et al. in France examined 20 anatomical landmarks in temporal bones using CBCT and multislice CT (MSCT), finding that CBCT offered qualitatively superior identification of structures like the footplate with a radiation dose 22 times lower than MSCT [12]. Our study complements these qualitative findings by showing no significant difference in quantitative measurements between CBCT and MDCT, reinforcing CBCT’s role as a low-dose alternative.

The lower radiation dose of CBCT, as supported by multiple studies [16][17][19], is a critical advantage, particularly for patients requiring repeated imaging, such as those with chronic otitis media or cholesteatoma. In the context of dental imaging, Shweel et al. reported that CBCT provided clearer visualization of odontogenic cysts and tumors compared to MDCT (22). While their study focused on dental structures, it supports CBCT’s high-resolution capabilities, which are applicable to the temporal bone’s fine bony details. Collectively, these studies confirm CBCT’s reliability for otologic imaging.

## Conclusion

This study confirms the potential applicability of CBCT for imaging footplate thickness. CBCT provides equivalent results to MDCT, but the radiation dose is significantly reduced. CBCT is also less expensive than MDCT. Thus, it provides favorable results in cost-effective analyses (25). This study recommends the use of low-resolution CBCT due to the lower dose and the lack of significant difference in measurement between the 3 methods.

## Conflict of Interest

None.
